# Impaired Cross-Modal Inhibition in Alzheimer Disease

**DOI:** 10.1371/journal.pmed.0020288

**Published:** 2005-09-20

**Authors:** Alexander Drzezga, Timo Grimmer, Martin Peller, Marc Wermke, Hartwig Siebner, Josef P Rauschecker, Markus Schwaiger, Alexander Kurz

**Affiliations:** **1**Department of Nuclear Medicine, Technische Universität München, Munich, Germany,; **2**Department of Psychiatry and Psychotherapy, Technische Universität München, Munich, Germany; **3**Department of Neurology, Christian-Albrechts-Universität, Kiel, Germany,; **4**Department of Physiology and Biophysics, Georgetown University Medical Center, Washington, District of Columbia, United States of America; University of California at Los AngelesUnited States of America

## Abstract

**Background:**

Successful cognitive performance depends not only on the activation of specific neuronal networks but also on selective suppression of task-irrelevant modalities, i.e., deactivation of non-required cerebral regions. This ability to suppress the activation of specific brain regions has, to our knowledge, never been systematically evaluated in patients with Alzheimer disease (AD). The aim of the current study was to evaluate both cerebral activation and deactivation in (1) healthy volunteers, (2) patients with mild cognitive impairment (MCI) who are at risk for AD, and (3) patients with moderate AD during active navigation, representing a cognitive task typically affected in AD.

**Methods and Findings:**

Changes in regional cerebral blood flow (rCBF) were assessed with PET imaging during an active navigation task in a 3D virtual-reality environment. The task was based on visual cues exclusively; no auditory cues were provided. Age-matched groups of healthy individuals, patients with MCI, and patients with AD were examined. Specific differences in the activation patterns were observed in the three groups, with stronger activation of cerebellar portions and visual association cortex in controls and stronger activation of primary visual and frontal cortical areas in patients with MCI and AD. Highly significant bilateral decrease of rCBF in task-irrelevant auditory cortical regions was detected in healthy individuals during performance of the task. This rCBF decrease was interpreted as a cross-modal inhibitory effect. It was diminished in patients with MCI and completely absent in patients with AD. A regression analysis across all individuals revealed a clear positive relation between cognitive status (mini mental state examination score) and the extent of auditory cortical deactivation.

**Conclusion:**

During active navigation, a high level of movement automation and an involvement of higher-order cerebral association functions were observed in healthy controls. Conversely, in patients with MCI and AD, increased cognitive effort and attention towards movement planning, as well as stronger involvement of lower-order cerebral systems, was found. Successful cognitive performance in healthy individuals is associated with deactivation of task-irrelevant cerebral regions, whereas the development of AD appears to be characterized by a progressive impairment of cross-modal cerebral deactivation functions. These changes may cause the generally decreased ability of patients with AD to direct attention primarily to the relevant cognitive modality.

## Abstract 2

When performing a navigation task with exclusively visual clues, healthy individuals de-activate areas of the brain involved in hearing. In individuals with Alzheimer Disease, no deactivation is seen.

## Introduction

It is a common hypothesis that information processing capacities of the brain are generally limited. Therefore, these capacities must be focused on the relevant sensory features and modalities. Modality-dependent selective attention mechanisms not only rely on the activation of specific neuronal networks, but also on selective suppression of task-irrelevant modalities, i.e., inhibition of less involved cortical areas [1]. This mechanism has been referred to as “cross-modal” inhibition. The underlying hypothesis is supported by several studies using neuroimaging tools. Particularly, cross-modal auditory/visual deactivation has been demonstrated repeatedly in healthy individuals [2–4].

In patients with Alzheimer disease (AD), multiple attention- and perception-related cognitive deficits are well-known. Functional neuroimaging studies offer a chance to evaluate the functional correlates of these deficits and to identify compensatory strategies. In some studies, changes in cerebral activation patterns have already been demonstrated in patients with AD [5,6]. However, to our knowledge, it has never been evaluated whether altered cerebral inhibitory processes are also involved in the cognitive deficits typically present in neurodegenerative disorders. Spatial navigation is among the first cognitive functions to be impaired in AD, leading to severe limitations in independent living. Perceptual and attention-related functions are particularly essential for this complex cognitive process, and previous neuroimaging studies have demonstrated extended activation of multi-modal cerebral systems in healthy individuals during navigational tasks [7]. However, information on functional cerebral changes during navigation in patients with AD is extremely limited.

In the present study, we examined changes of regional cerebral blood flow (rCBF) during active navigation in terms of increases (activation) and decreases (deactivation). We included healthy volunteers, patients with early AD, and patients with mild cognitive impairment (MCI). Patients with MCI were included because they represent a risk population for AD [8]. We selected a navigation task based on visual cues in a specially designed 3D virtual-reality (VR) environment, in order to simulate a real-life situation. All individuals had to navigate from a predefined starting point to a destination, and performance was measured in terms of time required to reach the end point. The purpose of the study was to identify differences between healthy individuals and the patient groups regarding cerebral activation and, particularly, cerebral deactivation during navigation.

## Methods

### Participants

Participants were recruited prospectively at the university outpatient clinic for memory research. Prior to starting the actual study protocol, all participants underwent an extensive evaluation including the following: (1) F18 FDG PET imaging (Siemens ECAT HR+ PET scanner, CTI, Knoxville, Tennessee, United States) and subsequent data analysis (NEUROSTAT, University of Michigan, Ann Arbor, Michigan, United States) to assess cerebral metabolic patterns [9,10]; (2) structural MRI (1.5 Siemens “Magnetom Symphony”) for exclusion of anatomic abnormalities, vascular pathology, or major atrophy; and (3) extended neuropsychological examination (CERAD-NP [Consortium to Establish a Registry for Alzheimer's Disease Neuropsychological battery], CDR [clinical dementia rating], GDS [geriatric depression scale], and ADL [activities of daily living]). Subsequently, participants were subdivided into three groups: (1) healthy volunteers, (2) patients with MCI, and (3) patients with moderate AD. This subdivision was performed according to established diagnostic criteria regarding neuropsychology, clinical examination and F-18 FDG PET results [9]. Based on the results of a previous study, patients with MCI but without any characteristic abnormalities in F-18 FDG PET and healthy participants who did show suspicious PET findings were excluded [9]. Using this approach, we attempted to enrich the MCI population with high-risk patients, in order to homogenize the groups and to exclude “healthy” controls with apparent brain pathology. Furthermore, participants with other neurological or psychiatric disorders or on medication with possible psychotropic effects were excluded. All participants were right-handed (assessed by the Edinburgh inventory; [11]). The study protocol was approved by the ethics committee of the Technische Universität München and the radiation protection authorities.

### Materials

An IDL-based VR environment computer system was developed, operating on an SGI O2 workstation (Silicon Graphics, Mountain View, California, United States) for application in the PET scanner. A monitor was installed on a platform in front of the PET gantry, allowing direct view from the scanner. Three-dimensional stereoscopic perception was ensured using shutter glasses (StereoGraphics, San Rafael, California, United States). A SpaceMouse (SpaceBall, Labtec, Vancouver, Washington, United States) fixed laterally at the scanner allowed free movement within the system with minimal motion of the hand (Figure 1A). Based on this VR system, two different types of virtual environments were designed: (1) the control condition, constituted by a never-ending rectangular pathway without predetermined start or end points or turn-offs, and (2) the test condition, consisting of a trail across a sequence of four rooms in a complex spatial arrangement with additional exits, enabling false turn-offs into blind rooms (Figure 1B). Both conditions resembled the inside of a simple building. A plain design was selected and the general features of the environment (color, texture, and size) were kept identical to ensure an overall comparable visual impression and to avoid unnecessary distraction of the participants and contamination of the cognitive process of navigation. The entire system was evaluated and proved applicable in patients with AD in previous experiments [12].

**Figure 1:**
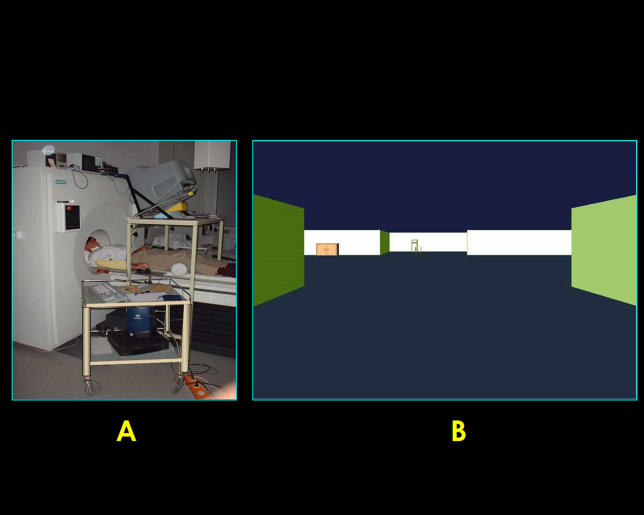
Experimental Setup (A) Experimental setup, showing a participant in the PET scanner during performance of a navigation task in the VR environment. (B) Snapshot of the visual impression of the test condition in the virtual environment at the start point of the navigation task.

### Procedure

Before the actual start of the scans, all participants were trained in the virtual environment outside of the scanner in order to familiarize them with the levels and handling of the system. Participants who were not able to perceive the virtual environment three-dimensionally or to operate the navigation system properly were excluded from the study. The performance of all participants was also observed during the entire PET examination by a study coordinator, to ensure correct transaction of every single scan. Participants performed the task in complete silence; the study coordinator was quietly watching the patient during the entire examination to report potential contamination with noise and to supervise the patient without the need of verbal interaction. Patients were instructed not to talk during the examination except in case of emergency. In the control condition, participants were instructed to steer along the never-ending pathway, thus no actual navigation was required but visual and motor demands were similar to the test condition. In the test condition, participants were asked to find their way from a start point to a predefined destination point. The two different conditions of the VR environment were presented in the scanner in a randomized order (4× control condition and 8× test condition). During the test conditions, performance (in terms of time required to reach the destination) was recorded.

### O-15 Water PET Acquisition

A Siemens ECAT HR+ PET scanner was used for O-15 PET measurements (3D mode; total axial field of view, 15.5 cm). For each scan, 350 MBq of O-15 water was injected in a slow intravenous bolus after start of the VR paradigm using an infusion pump. Data acquisition was triggered by the peak of count rate. Each scan lasted 50 s for the measurement of rCBF. Twelve scans were performed in each participant. Attenuation-corrected data were reconstructed (63 slices; 128 × 128 pixel matrix; pixel size, 2.0 mm; plane separation, 2.42 mm).

### Data Analysis

Statistical parametric mapping software (SPM 99, Wellcome Department of Cognitive Neurology, London, United Kingdom) was used for image realignment, transformation into standard stereotactic space, smoothing, and statistical analysis, resulting in 26 planes (pixel size, 2 × 2 mm; interplane distance, 4 mm), as previously described [13].

The effect of global differences in cerebral blood flow was removed by treating global activity as a confounding variable and using participant-specific scaling to a nominal grand mean global activity of 50 ml/100 g/min. Thus, data were adjusted for the global mean (rCBF), taking into account the repetitions across participants. The adjusted voxel values were then used for further statistical analysis [14].

The statistical analysis was performed according to the general linear model and the theory of Gaussian fields at each and every voxel using a mixed-effects model [13,15,16]. The resulting statistical parametric maps based on the *t* statistic were subsequently transformed into normally distributed statistical parametric *(Z)* maps [13].

A network of expected cortical activations was predefined based on previous publications on cerebral activation during visual spatial processing and active navigation [7,17–19]. This network included cerebral regions belonging to the ventral and dorsal streams of the visual system, such as primary and secondary visual cortex, inferotemporal cortex, ventrolateral prefrontal cortex, posterior parietal cortex, and dorsolateral prefrontal cortex. In addition, cerebral regions traditionally linked to spatial orientation were included in the network, such as the hippocampus (allocentric mapping, recognition of landmarks), posterior and superior parietal cortex (spatial attention, egocentric mapping, and optic flow processing; see above), and posterior cingulate cortex (spatial orientation). Finally, as the task required active movement, we expected activation of motor-associated systems such as premotor cortex, as well as supplementary and primary motor cortex (associated with movement planning and execution, respectively). Stereotactic coordinates of these anatomic regions were selected according to corresponding Brodmann areas (BAs) in the atlas of Talairach and Tournoux [20].

As mentioned previously, we expected to find cross-modal deactivation of auditory cortical areas. Therefore, as for cerebral activations, a network regarding deactivations was predefined based on previous data on the extent of auditory cortical areas [21,22] and on information about cross-modal deactivation of auditory areas during visually dominated tasks [2,23]. This network included BAs 41, 42, 21, and 22 in both hemispheres, representing primary auditory cortex (A1) and adjacent belt areas that have been linked to auditory associative functions [22,24]. Corresponding to former studies, analysis was mainly restricted to these areas [25]. Regarding the interindividual and interhemispheric variability of the location of the auditory cortical areas, we defined three-dimensional volumes independently for each hemisphere representing the probabilistic location of the primary auditory cortex according to Penhune et al. [21]. These volumes were transferred into the stereotactic reference system, to allow for correct anatomical assignment of the results. Within the predefined expected cortical activation and deactivation networks, all statistical results were based on a single-voxel *z*-threshold corresponding to *p* < 0.001, uncorrected for multiple comparisons. To our knowledge, no conclusive previous information on cerebral deactivation during visually guided navigation exists. Thus, only voxels surviving false discovery rate (FDR) correction for the entire volume at *p* < 0.05 were accepted in the statistical analysis of rCBF decrease between conditions within groups, in order to avoid false-positive results [26].

First the rCBF changes between the control and test conditions were examined in each of the different groups (control individuals, patients with MCI, and patients with AD). For the assessment of differences of rCBF changes between the groups, a “difference of differences” analysis was used. In all group analyses, the rating of navigation performance of participants (time required for passage from start to end point of the labyrinth) was used as a covariate of no interest, to diminish the possible effects due to individual differences of performance or subjective experience of the paradigm. Finally, a voxel-based linear regression analysis of cognitive performance (as measured with the mini mental state examination [MMSE]) and regional cerebral deactivation was carried out in the entire population, containing the participants from all three groups. We generated subtraction images of the individual patients (control condition) and put these images in relation to the MMSE scores of the patients, using a “covariate only” analysis. This analysis tested for voxels in the brain in which task-related deactivations showed a significant linear relationship with the MMSE scores. An a priori hypothesis for the location of the probable linear relation was defined for this analysis, based on the previously identified deactivation foci, and an uncorrected threshold of *p* < 0.001 was applied. We limited the linear regression analysis to clusters within the A1 (transverse gyrus of Heschl [TGH]). Additionally, a small volume correction was performed within a sphere of 20-mm radius, centered on the maximum deactivation in the TGH. The results of all analyses (maxima of the activation foci) were reported with the respective standard stereotactic coordinates according to Talairach and Tournoux [20].

## Results

Based on the inclusion criteria, 32 participants were recruited and allocated to the three predefined age-matched groups: (1) 11 healthy volunteers (three female), (2) 11 patients with MCI (four female), and (3) ten patients with moderate AD (four female) (Table 1). No significant difference in age was detected (healthy versus MCI, *p* = 0.50 [95% confidence interval [CI], −5.75 to 11.39]; healthy versus AD, *p* = 0.43 [95% CI, −5.63 to 12.78]; MCI versus AD, *p* = 0.85 [95% CI, −7.54 to 9.07]). Regarding cognitive function, there was no significant difference in mean MMSE scores between control individuals and patients with MCI. However, patients with AD yielded significantly lower scores than controls (healthy versus MCI, *p* = 0.06 [95% CI, −2.97 to 0.06]; healthy versus AD, *p* < 0.001 [95% CI, −5.84 to −2.39]; MCI versus AD, *p* = 0.02 [95% CI, −4.87 to −0.46]).

**Table 1:**
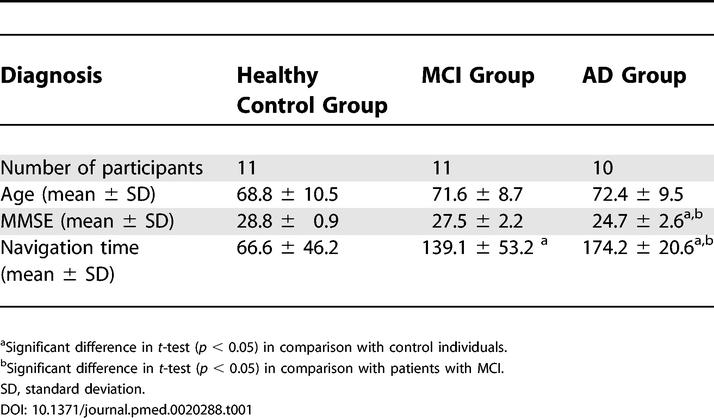
Participant Characteristics

### Navigation Performance

Each of the 32 participants underwent a total of 12 O-15 water activation PET scans during active navigation and control conditions in the virtual environment, resulting in a total of 384 scans. All participants were able to understand the task and cope with the test paradigm requirements satisfactorily. The mean navigation time needed by the participants to reach the destination from the start point during test condition differed considerably between the groups (Table 1). In patients with MCI, performance was significantly impaired when compared to control individuals; still, they performed significantly better in the navigation task than patients with AD. The latter required significantly more time for accomplishment of the task, as compared to healthy control indivduals and patients with MCI (healthy versus MCI, *p* < 0.001 [95% CI, −87.44 to −57.54]; healthy versus AD, *p* < 0.001 [95% CI, −118.68 to −96.50]; MCI versus AD, *p* < 0.001 [95% CI, −47.64 to −22.55]).

### Cerebral Activation

In the statistical group comparison between the control condition and navigation condition we found a significant increase in rCBF in a number of cortical regions associated with the predefined navigation network in all three groups (Table 2; see Figure 2). These increases included strong activation of posterior and superior parietal cortical areas, particularly the precuneus. Furthermore, all groups showed activation of visual areas. In the healthy group, activation of higher-order extrastriate visual areas (BA 19) was detected bilaterally. Less involvement of these areas in active navigation was found in patients with MCI (left hemisphere only), and none at all in patients with AD. Conversely, strong activation of primary visual and adjacent cortical areas (BA 17/18) was observed in patients with AD, to a lesser extent also in patients MCI, whereas no significant activation of these cerebral regions was detected in control indivduals. Motor activation included left sensorimotor cortex and supplementary motor cortex in the healthy control indivduals. Several cerebellar regions were activated during navigation in control individuals, whereas cerebellar activation was considerably less in patients with MCI and absent in patients with AD, and no activation of primary motor cortical areas was observed. However, activation of premotor and prefrontal areas (BA 8 and BA 10) was detected exclusively in patients with AD and MCI, respectively. Analysis of the difference of differences confirmed significantly stronger activation of cerebellar regions in healthy control individuals than in patients with MCI and AD, and stronger activation of extrastriatal visual areas than in patients with AD. In both the AD and MCI groups, the premotor and prefrontal cortical activations were found to be significantly stronger than in the healthy control group. Generally, the strongest differences were observed between patients with AD and control individuals, whereas patients with MCI showed specific similarities with both other groups. In none of the groups was any activation of auditory or auditory-associated cortical areas observed.

**Figure 2:**
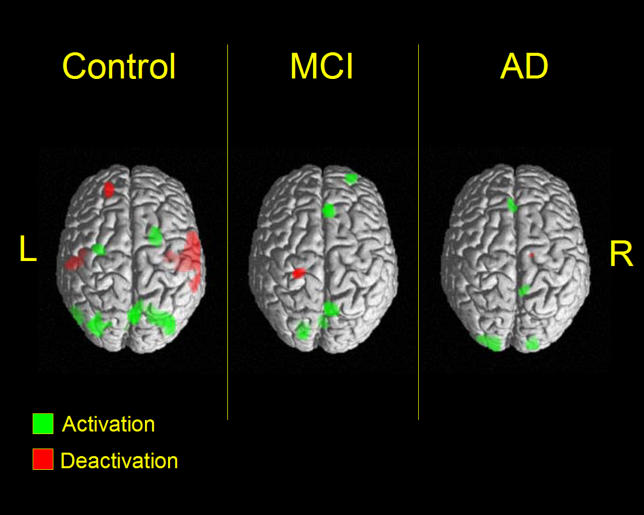
rCBF Changes in Control Individuals and Patients with MCI and AD during Navigation Task Results are surface-rendered and superimposed on a standard MRI template. Green indicates significant increase of rCBF, and red indicates significant decrease of rCBF during active navigation (results are displayed at *p* < 0.001).

**Table 2:**
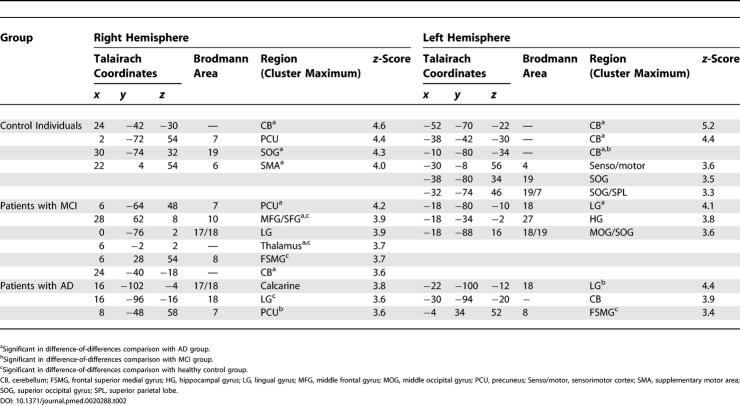
Activation of Cortical Areas during Navigation

### Cerebral Deactivation

In the statistical comparison between the control condition and navigation condition we found a significant decrease in rCBF in bilateral auditory cortical areas in the healthy control group during active navigation (Table 3; Figures 3 and 4A). This suppression of auditory-associated areas in healthy volunteers was demonstrated bilaterally, but with a predominance of the right hemisphere. A major portion of the deactivated area was located bilaterally within the predefined probability map of the A1, according to previously published criteria [21] (Figure 3). In addition, auditory cortex in the adjacent belt areas [22] was also deactivated. The strongest deactivation was found ventrolaterally to A1 in the right hemisphere, but deactivations were also observed in belt areas in the dorsal and caudal vicinity of A1 (Table 3; Figure 3).

**Figure 3:**
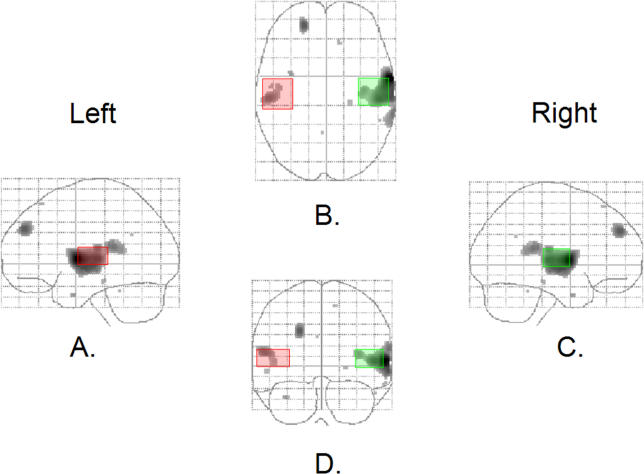
Auditory Cortical Deactivation Areas of significant deactivation in healthy volunteers (*p* < 0.001) during navigation are demonstrated in black on a glass-brain display. The probabilistic volume of A1 according to Penhune et al. [21] is outlined in green (right hemisphere) and red (left hemisphere). Aspects are (A) left lateral, (B) cranial, (C) right lateral, and (D) dorsal.

**Figure 4:**
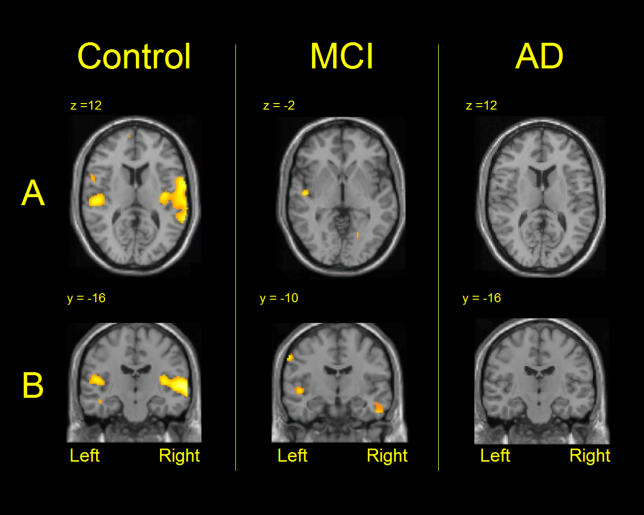
Deactivation in Control Individuals and Patients with MCI and AD Results are superimposed on a standard MRI template. Yellow indicates significant cerebral deactivations during active navigation: (A) axial slices, cranial aspect; (B) coronal slices, dorsal aspect (results are displayed at *p <* 0.005, for illustration purposes).

**Table 3:**
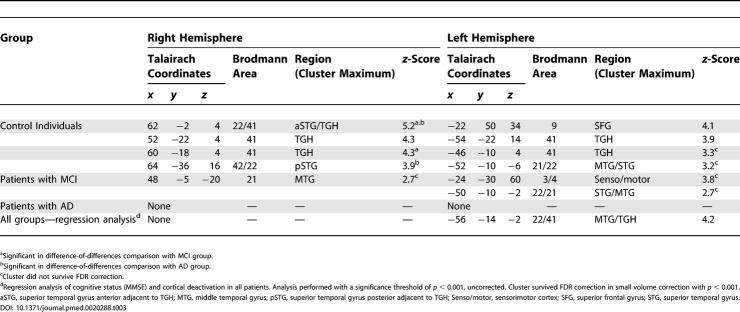
Deactivation of Cortical Areas during Navigation

Interestingly, only faint deactivation was observed in patients with MCI (Table 3; Figure 4B), and exclusively in auditory belt regions. These findings did not survive FDR correction for the entire volume. The difference-of-differences analysis confirmed that deactivation effects in right-hemispheric auditory cortical regions were significantly stronger in controls than in patients with MCI.

Moreover, patients with AD did not show any significant deactivation of task-irrelevant auditory cortical areas during visually based navigation (Table 3; Figure 4). Again, a difference-of-differences analysis confirmed that deactivation effects in right temporal auditory regions in controls were significantly stronger in control individuals than in patients with AD, whereas no significant difference between patients with MCI and AD was found (Table 3; Figure 4). Little extra-auditory deactivation was observed, and was seen exclusively in left superior prefrontal cortex (BA 9) of healthy participants and in left sensorimotor cortex (BA 3/4) of patients with MCI, with the latter effect not surviving the FDR correction.

### Linear Regression Analysis

A voxel-based regression analysis of regional cerebral deactivation with cognitive performance (as measured with the MMSE score) was performed, in order to identify a possible connection between the degree of overall cognitive impairment and the deactivation capabilities. In this analysis, a clear linear relation of the MMSE scores with deactivation of left auditory cortex (BA 41) was indeed detected (Table 3; Figure 5), pointing to a direct association of cognitive performance with cortical deactivation. This cluster survived FDR correction with a *p* < 0.001 in the small volume correction. No additional significant linear relation was observed. Additionally, we performed a correlation analysis using the MMSE scores and the regional adjusted rCBF response at the location with Talairach coordinates *x,* −56; *y,* −14; and *z,* −2. This analysis revealed a coefficient of correlation *r* of −0.67 (*p* < 0.001 [95% CI, −0.83 to −0.42]).

**Figure 5:**
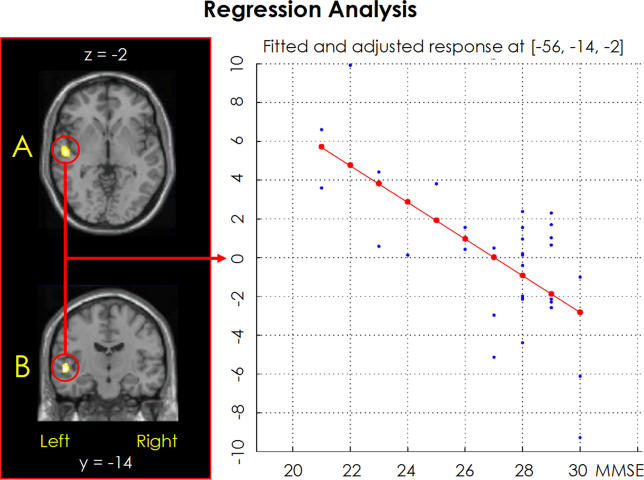
Positive Linear Regression of Auditory Cortical Deactivation with Cognitive Performance (MMSE) in All Participants Left: results are superimposed on a standard MRI template. Yellow indicates a significant relationship of cerebral deactivation with the MMSE: (A) axial slice, cranial aspect; (B) coronal slice, dorsal aspect (results are displayed at *p* < 0.005, for illustration purposes). Right: Regression analysis (red indicates regression line) of the fitted and adjusted rCBF response (blue points, arbitrary units) to active navigation in relation to the MMSE score at the position of the significant cluster (Talairach coordinates *x,* −56; *y,* −14; *z,* −2; *p* < 0.001, uncorrected).

## Discussion

### Activation of Cortical Regions during Visual Navigation

In previous studies, extended cerebral networks associated with human navigation have been identified [7,17,18]. In the current study we observed a regional increase of rCBF, interpreted as cerebral activation in several elements of these predefined networks, in three groups of individuals (healthy control individuals, patients with MCI, and patients with AD) during active navigation in a VR environment. Consistent with previous studies, strong activation of parietal cortical areas was found during the navigation task. Generally, a role of the parietal cortex in visual spatial functions including spatial attention is widely accepted [1,27]. Parietal cortical areas are part of the dorsal stream of the visual cortical system and as such are involved in the processing of spatial and motion information [28].

In a previous study on navigation in VR, activation of medial parietal areas was identified and associated with optic flow induced by egocentric movement [7]. The medial parietal activations found in our study conform to such functions. In addition, recent studies suggest a role for medial parietal cortex (in particular the precuneus) in episodic memory retrieval, which may also be required for navigation [29].

In addition to parietal cortex, activation of visual cortical regions was observed in all three groups. In the healthy group, extended activation of extrastriate cortex within bilateral BA 19 was detected, i.e., in a cortical region that contains the visual association areas V3–V5, which have been linked to higher-order visual functions, such as perception of movement and shape and form of moving objects [30]. Less involvement of these areas in active navigation was found in patients with MCI and none at all in patients with AD. Conversely, strong activation of primary visual cortex (BA 17) and adjacent BA 18 was observed in patients with AD, and to a lesser extent also in MCI, whereas no significant activation of these early visual regions was detected in control individuals. This suggests that activation induced by the increased cognitive demand of the navigation task is restricted to lower-order systems of the visual hierarchy in patients with MCI and AD and does not lead to recruitment of higher-order visual association areas as in control individuals. This may be a consequence either of ongoing neurodegenerative changes, disconnection phenomena, or a shift in attentional priority and is also consistent with previous observations [31].

In healthy individuals, increased activation of left primary sensorimotor cortex and paramedian premotor cortex (supplementary motor cortex) was observed during active navigation, consistent with a more resolute motor execution (performed with the right hand) than in the control condition. No comparable activation was observed in patients with MCI or AD, consistent with a more hesitant performance of the task in these groups. Furthermore, stronger and more extended activation of cerebellar areas was detected in healthy volunteers than in patients with MCI and AD. Increasing evidence has been provided for a role of the cerebellum in cognitive functions, including navigation [32], which could explain the activation detected in the current study. Additionally, a major role of the cerebellum in automation of executive functions is widely accepted [33]. It appears plausible that healthy participants employed a more automated strategy to solve the navigation task, whereas patients with AD, and even patients with MCI, shifted to a less automated, more attentionally demanding approach. This hypothesis is supported by the exclusive activation of prefrontal cortical regions in patients with MCI (BA 8 and BA 10) and AD (BA 8). Prefrontal cortical areas have been associated with executive functions such as planning, problem solving, and reasoning but also with spatial attention and working memory [2,34,35]. In particular, the region in superior prefrontal cortex approximately anterior to the frontal eye field, which was activated in both patient groups, is thought to be specialized for spatial working memory [36,37].

A general limitation of the current study may be that an interaction between participant group and control versus test condition can not be completely excluded, as we could not measure performance of participants during control condition. However, the control condition was only used as a baseline (for correction of visual and sensorimotor effects) and no actual “navigation” was required.

### Deactivation of Auditory Regions during Visual Navigation in Healthy Control Individuals

In the virtual navigation task used in our study, all external information regarding current localization was based on visual input; auditory cues were neither provided nor required. Therefore, we expected cross-modal inhibition of those cortical regions that are typically associated with cerebral processing of auditory information. Indeed, we found a striking decrease in rCBF in bilateral auditory cortical areas in the healthy control group during active navigation.

Corresponding to many previous studies we interpreted this regional decrease in cerebral rCBF as local cerebral deactivation [3,4,38]. In a recent study, regional decrease of fMRI BOLD signal was explicitly associated with true cortical deactivation, which supports our interpretation [39]. Even if the observed rCBF changes in the current study represented only regions spared from global activation effects, they would suggest cerebral inhibitory effects, justifying the term “deactivation.” The deactivation effect may also involve inhibitory neurons, but considering the small number of inhibitory cortical neurons in general (∼10%), a contribution to the observed results in our study is not probable [40].

Interference between visual and auditory information in the brain is well-documented [25], and cross-modal inhibition between the two modalities has been discussed in prior studies [23]. In particular, it must be taken into account that all task-relevant information in our study was derived from the visual stimuli in the virtual environment, whereas any auditory signals would have originated from the real local environment and, thus, would have been irrelevant or even misleading. Consequently, in our study, a suppression of auditory-associated areas in healthy volunteers was demonstrated predominantly in the right hemisphere, which is widely thought to be “specialized” for spatial attention [27,41], including in the auditory domain [42]. A major portion of the deactivated areas was located bilaterally within A1, as defined previously [21]. In addition, auditory cortex in the adjacent belt areas was deactivated [22,43]. The maximal deactivation was found anterolateral to A1, in a region that has been associated in animal and human studies with the identification of auditory objects [22,44,45]. However, deactivation was also observed posterior to A1 in regions that have been associated with the processing of auditory spatial information [22,42,46]. It appears, therefore, that in healthy individuals interference from conflicting auditory signals is minimized during processing of a visual navigation task by suppressing auditory activity at relatively early stages of cortical processing.

### Absence of Auditory Deactivation in Patients with AD and MCI

The loss of navigation abilities represents one of the most disabling cognitive impairments in AD. The tendency to become lost can be found in almost all patients with AD in the course of the disease. Accordingly, in our study a significantly impaired performance in the navigation task was observed in patients with AD as compared to healthy control individuals and patients with MCI.

Interestingly, in contrast to healthy control individuals, patients with AD did not show any significant deactivation of task-irrelevant auditory cortical areas during visually based navigation. Often, the loss of orientation in AD has been associated with impaired memory function [47]. More recently, however, navigation impairment in AD has been attributed primarily to impaired perceptual abilities, such as optic flow discrimination, associated with constraints to attention and disturbed multi-sensory integration [48–50]. Perception of any type is inseparably related to attention. Multiple attentional deficits, concerning spatial and selective attention, as well as the inability to shift attention across levels of perceptual organization, are well-documented in patients with AD [51–54]. The principle of suppression of irrelevant input has been discussed as a mechanism essential for selective attention to a certain modality [1].

Therefore, the impaired visual navigation performance of patients with AD may be related to the striking absence of auditory deactivation observed in these individuals. Parasuraman et al. associated the abnormal disengagement/shifting abilities in patients with AD with a possible dysfunction of cortico-cortical networks [53]. This is of particular interest, as a dysfunction in cortical regions that are not primarily affected by the Alzheimer pathology has been detected in the current study [55].

### Absence of Cross-Modal Deactivation as an Early Indicator of AD

Unfortunately, spatial disorientation is not a late symptom of AD, but has been shown to be one of the first signs of the disease. Impaired navigation abilities are observed in patients with MCI [56]. Like in patients with AD, perceptual deficits, such as impaired visual motion processing, have been associated with poorer performance in spatial navigation tests in patients with MCI [56]. Again, the affected perceptual functions are tightly associated with attention. Similarly to in patients with AD, attentional deficits have been documented in patients with MCI [48,57].

In our study, patients with MCI still performed significantly better than patients with AD in the navigation task; however, their performance was significantly impaired compared to that of control individuals. At the same time, only faint deactivation in auditory belt regions was observed in MCI patients. Thus, impaired attention-focusing properties may also be involved in disorientation in MCI. Considering that the examined patients with MCI did not yet fulfill criteria for dementia in neuropsychological assessment, this observation is particularly interesting, and it indicates that reduced deactivation of auditory areas in a visual navigation task could be used as an early indicator of AD.

An ongoing follow-up study using the same examination paradigm as in the current study confirms this hypothesis. A preliminary analysis of baseline data from patients with MCI (*n* = 15) revealed significant auditory deactivation (BA 41; Talairach coordinates *x,* 62; *y*, −4; and *z,* 4) exclusively in the subgroup of patients with MCI who remained cognitively stable (*n* = 8) within the observation period (2 y). In contrast, the group of patients who proceeded to AD (*n* = 7) during this time, showed no auditory deactivation at the initial evaluation. Differences in cerebral activation patterns have been previously considered to be useful for early diagnosis of AD [58]; the present results indicate that it may be even more effective to direct one's attention to changes in cerebral deactivation patterns.

### Other Cortical Deactivations

Hardly any extra-auditory deactivation was observed in any of the groups. Exclusively in healthy participants a deactivation of a region located in the prefrontal cortex (BA 9) was observed in a region that has been associated with visuo-spatial attention, particulary to stimuli at selected peripheral locations [35]. Since the navigation task in the current study required attention directed to the center of gaze, a deactivation of this periphery-oriented attention area appears economical. The focal rCBF decrease in left sensorimotor cortex in patients with MCI did not survive FDR correction and may be the result of less resolute motor execution in the navigation condition than in the control condition.

### Relation of Auditory Deactivation to Cognitive Performance

In addition to statistical group comparison, we performed a voxel-based regression analysis in the entire study population, to further evaluate a possible association of cognitive function with the deactivation properties. In this analysis, individual cognitive performance (MMSE score) across all participants was shown to be directly associated with the intensity of cerebral deactivation in the auditory cortex. This result is highly specific, considering that an association of cognitive function with rCBF changes was not observed in any other cerebral region. This implies that physiologic cerebral deactivation capabilities are indeed essential for normal brain function and appear to be progressively affected in ongoing neurodegeneration. Interestingly, the identified linear regression effect was only significant in the left hemisphere, i.e., the dominant side of the brain in our purely right-handed population. Thus, the changes in deactivation functions in relation to overall cognitive impairment may be most clearly expressed in the dominant hemisphere.

Impaired cortical inhibitory capabilities in patients with AD appear plausible for two reasons. First, a stronger vulnerability of the neocortical inhibitory system has often been suggested. Second, recent studies were able to demonstrate cortical disinhibition in AD and could relate it to a cholinergic deficit [59,60].

### Conclusions

The results of this study indicate that altered deactivation patterns must be taken into account in activation studies comparing effects in patient groups versus control individuals, in order to avoid misinterpretation of differences. We have demonstrated that navigation based on visual cues induces a deactivation of auditory cortical areas in healthy individuals. These deactivation effects are impaired in patients with MCI and absent in patients with AD, pointing to a progressive inability to tune out irrelevant input and to focus attention on the task-relevant modalities. Thus, the orientation disability in the outside world seen in patients with AD may in fact be partially based on the inability to selectively orient spatial attention to task-relevant internal representations of perceptual stimuli.

**Patient Summary**

**Background**

Problems with finding one's way are one of the early signs of Alzheimer disease (AD). Researchers can measure how good people are at “spatial orientation” by asking them to solve navigation tasks on the computer. These are similar to virtual-reality video games in which one needs to find one's way based on a set of cues. Using brain imaging technology, scientists can then observe what is going on in the brains of people while they solve such navigation tasks.

**Why Was This Study Done?**

Scientists used to think of AD as mostly a disease of memory loss, and loss of orientation was considered to be mostly a memory problem. More recently, however, navigation problems have been linked to problems with perception and with paying attention. In this context, the researchers wanted to compare the brain activities in people with and without AD while they completed such navigation tasks. They were looking for which areas of the brain were activated, and also whether others (which had nothing to do with the navigation task) were deactivated. The idea was that maybe to do the task well, individuals need to both activate relevant areas of the brain and also deactivate others to be able to focus overall brain activity on processes that would help with the task at hand.

**What Did the Researchers Do and Find?**

They studied brain activity in three groups of people while the individuals solved a navigation task. The first group consisted of 11 healthy individuals, the second of ten individuals with mild cognitive impairment, and the third of 11 individuals with AD. The cues that guided them through the navigation task were exclusively visual, and all of the participants were able to understand the cues. They found that the activation patterns were different between the three groups. The healthy individuals showed more activity in parts of the brain thought to be involved in processing visual cues, whereas the other two groups showed stronger activation in the parts involved with visual perception. Moreover, the researchers found differences in the deactivation patterns, mostly in the area of the brain that deals with sound and hearing. In healthy individuals these areas shut down to some extent during the visual navigation task, but this effect was diminished in individuals with mild cognitive impairment, and individuals with AD showed no signs of deactivation in these “task-irrelevant” areas.

**What Does This Mean?**

This study suggests that in addition to difficulty integrating complex information, inability to focus activity in the relevant parts of the brain might be contributing to some of the orientation problems in patients with AD. It is not clear whether cognitive therapy could strengthen the ability to “focus one's brain,” but it seems an idea worth pursuing. The researchers also suggest that reduced deactivation of auditory areas in visual navigation tasks might be useful as an early indicator of AD, and they are currently doing studies to test this idea.

**Additional Online Information**

The following Web sites contain information on AD in general and describe some of the changes in the brains of patients with AD.

Alzheimer Research Forum:

http://www.alzforum.org/home.asp

US National Institute on Aging:

http://www.nia.nih.gov/

Alzheimer's Disease Education and Referral Center (search for “brain imaging”):

http://www.alzheimers.org/pubs/adfact.html

Alzheimer's Association (search for “brain imaging”):

http://www.alz.org/

US National Institute of Neurological Disorders and Stroke's Web page on AD:

http://www.ninds.nih.gov/disorders/alzheimersdisease/alzheimersdisease.htm

## Abbreviations

A1: primary auditory cortex
AD: Alzheimer disease
BA: Brodmann area
CI: confidence interval
FDR: false discovery rate
MCI: mild cognitive impairment
MMSE: mini mental state examination
rCBF: regional cerebral blood flow
TGH: transverse gyrus of Heschl
VR: virtual reality
